# Compliance patterns in adopting sustainability practices: A cluster analysis of oil palm producers in Colombia

**DOI:** 10.1038/s41598-026-43888-9

**Published:** 2026-03-13

**Authors:** Julián F. Becerra-Encinales, Brayan Rodríguez, Eloína Mesa-Fuquen, Paloma Bernal-Hernández, Alexandre P. Cooman, Luis H. Reyes, Juan C. Cruz

**Affiliations:** 1 Colombian Oil Palm Research Center Corporation-Cenipalma , Bogotá, 111121 Colombia; 2 Department of Business Administration, Pontificia Universidad Javeriana, Bogotá, 110911 DC Colombia; 3 School of Engineering, Los Andes University, Bogotá, 111711 DC Colombia; 4 Interdisciplinary Centre of Development Studies - CIDER, Los Andes University, Bogotá, 111711 DC Colombia

**Keywords:** Cluster analysis, Sustainability index, Sustainable agriculture, Agricultural extension, Technological adoption, Oil palm, Environmental sciences, Environmental social sciences, Environmental studies

## Abstract

This study analyzes the adoption patterns of sustainability practices among oil palm producers in Colombia using cluster analysis of the Sustainability Index (SI). Employing advanced data mining algorithms, including K-means and Ward’s method, producers were grouped according to their compliance with sustainable practices at national and regional levels. The analysis revealed ten distinct producer typologies, ranging from “Advanced adopters” to “Lagging adopters” in economic, environmental, and social sustainability dimensions. Key factors influencing cluster formation included palm-growing area, producer scale, age, and gender. At the same time, the Palm Nucleus (organizational model for fruit commercialization) emerged as a significant variable at the regional level. Our findings challenge the conventional notion that adoption of sustainability practices is primarily scale-dependent, showing that contextual factors play a crucial role across all producer categories. This research underscores the importance of more nuanced, context-specific technological extension strategies. By providing a comprehensive understanding of adoption patterns, this study contributes to developing tailored interventions that can effectively promote sustainable practices in the Colombian oil palm sector, regardless of producer characteristics.

**Supplementary Information:**

The online version contains supplementary material available at 10.1038/s41598-026-43888-9.

## Introduction

In recent decades, sustainability has become a paramount concern in agricultural production, driven by growing consumer demand for environmentally and socially responsible products^1^. This trend is particularly evident in the oil palm sector, where sustainability certifications have become increasingly important for market access and differentiation. According to Hainmueller et al. (2015)^2^, the average annual growth rate in demand for Fair Trade-certified products in the United States exceeded 40% between 1999 and 2008, highlighting an early shift in consumer preferences toward sustainably produced goods. More recent empirical studies confirm that demand for certified, ethically sourced, and environmentally responsible agricultural products has continued to grow over the past decade. For example, Gagliardi et al. (2025)^3^ and Merbah and Benito-Hernández (2024)^4^ show that consumers are willing to pay significant price premiums for sustainability certification labels in agri-food markets. Similarly, Aminravan et al. (2025)^5^ and Zhan et al. (2025)^6^ demonstrate that consumer preferences for eco-labeled foods are increasingly influenced by trust in certification schemes and environmental awareness. These findings reinforce the relevance of sustainability certification as a market-driven mechanism for product differentiation and competitiveness in global food systems.

Agricultural exploitation framed in sustainability seeks to reduce the environmental impact of productivity while enhancing the quality of life for producers^7^. This approach is evidenced by certification companies that employ parameters or indices to measure compliance with standardized practices, evaluating and supervising to certify the sustainability of production systems. Garbely & Steiner (2023)^1^ argue that an agro-industrial system that responsibly integrates economic, environmental, and social aspects can significantly contribute to food security and poverty reduction. However, developing countries face particular challenges, including knowledge limitations, lack of specialized inputs, and the need for substantial investments in sophisticated equipment^8,9^.

In addition, sustainability certification in agriculture poses challenges in adopting practices, technologies, and innovations necessary for compliance with defined standards, commonly measured through Sustainability Indices (SI)^10^. Liu et al. (2021)^11^ and Salehi et al. (2021)^12^ point out that achieving potential productivity within a sustainability framework is particularly challenging in developing countries, especially when there is no well-developed pluralistic extension services system. Liu et al. (2021)^11^ emphasize that extension services have a statistically significant and strongly correlated relationship with the technical efficiency of agricultural operations and that different extension sources have an inconsistent impact on it. Adoption levels are often affected by farmers’ abilities and willingness, attributes of innovations and practices, and contextual factors (e.g. policy, environmental and market settings) limiting access to or applicability of technologies^13–15^, negatively impacting productivity, with small-scale producers bearing the brunt of these challenges^16–20^.

Mwangi & Kariuki (2015)^21^ emphasize that sustainability certification processes should be accompanied by technology extension systems for dissemination, transfer, and technical assistance to foster the adoption of sustainable practices^7,8^. These extension models must recognize producers’ needs and adapt tactics, transfer methodologies, and local context management approaches to overcome economic, environmental, and social barriers^22–27^. Such models should be inclusive, accommodating all producers regardless of their socio-economic characteristics^28–32^.

**Fig. 1:**
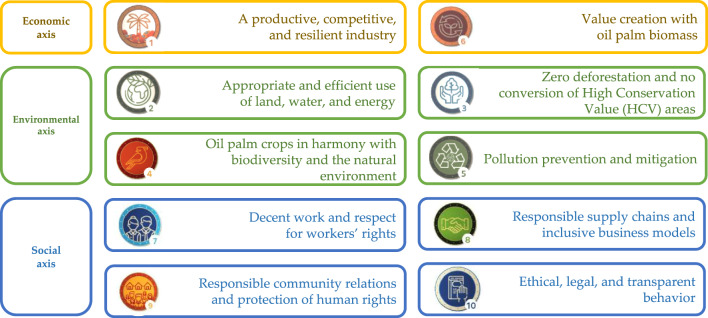
Sustainable Colombian Palm Oil Principles. Source APSColombia Corporation 2024^34^. In the yellow box, the economic axis of the SI is shown, along with its two corresponding principles. In the green box, the environmental axis of the SI is presented, with its four corresponding principles. Finally, in the blue box, the social axis of the SI is displayed, along with its four corresponding sustainability principles.

In response to this evolving sustainability landscape, Colombia’s oil palm sector has been implementing the “Palm Oil Sector Sustainability Strategy” and the “Sustainable Palm Oil Program of Colombia - APSColombia” since 2018, aiming to generate added value in palm oil production^33^. Fedepalma (2023)^33^ asserts that sustainability in the palm oil value and supply chain guarantees a unique and differentiated product. This sustainable origin is achieved through compliance with the “10 sustainability principles” of the APSColombia program, ensuring sustainable production of Colombian palm oil aligned with economic, environmental, and social axes of sustainability (Fig. 1).

The APSColombia program has significantly benefited the Colombian oil palm sector by consolidating its reputation for sustainable production. This has been crucial in countering international “No Palm Oil” campaigns, which arose due to deforestation and human rights violations in Southeast Asian producing countries^34^. Fedepalma et al. (2023)^35^ report that this initiative has helped overcome market access barriers and align with European consumer preferences for environmentally and socially sustainable products. As a result, nearly 30% of Colombia’s palm oil production now carries international sustainability certification, achieved by implementing targeted extension models that address sustainability gaps. These efforts position Colombia as a global leader in differentiating environmental, social, and economic practices and in promoting development that responds to market trends favoring sustainable products^33–35^.

Colombian oil palm producers face multiple barriers to sustainability adoption, including limited technical assistance coverage, variability in environmental regulations across regions, unequal access to financial capital, and heterogeneous organizational structures such as Palm Nuclei. These constraints translate into uneven compliance with sustainable practices, with some producers advancing rapidly while others lag significantly. Understanding these adoption patterns is essential to designing targeted extension and policy interventions that close gaps and avoid one-size-fits-all approaches. This study contributes to addressing this challenge by identifying producer typologies based on compliance behavior across the SI’s three sustainability axes.

In this context, the Sustainability Index (SI) emerged as a robust tool for measuring and monitoring sustainability in Colombian oil palm cultivation. It assesses the adoption of 79 practices, categorized into 29 issues, which align with the ten principles and three axes of sustainability illustrated in Fig. 1. See Supplementary Information (Appendix A, Table A1). The SI´s primary objectives are to quantify compliance and promote the adoption of best practices, technologies, and innovations that enhance the sustainability of oil palm cultivation in Colombia^33,34^.

The Sustainability Index (SI) used in this study is the only instrument in Colombia designed collaboratively by Fedepalma, Cenipalma, APSColombia, and environmental and labor authorities to integrate economic, environmental, and social principles into a single weighted metric. Unlike certification schemes such as RSPO, which focus primarily on compliance audits, the SI provides granular, practice-level information that supports decision-making at the farm and organizational levels. Its structure—comprising 79 practices across 29 issues and 10 sustainability principles—was developed through expert consultation and validated through pilot testing with more than 300 producers between 2018 and 2020. These features make the SI a robust tool for monitoring adoption patterns and identifying gaps across sustainability dimensions in the Colombian context.

In Colombia, Palm Nuclei act as coordination hubs linking producers with technical assistance, fruit commercialization, and sustainability programs. Their institutional capacity varies across regions, shaping producers’ access to training, governance structures, and pathways for sustainability adoption. Given their relevance for interpreting SI compliance patterns, their role is briefly introduced here and elaborated further in the Methods.

**Fig. 2:**
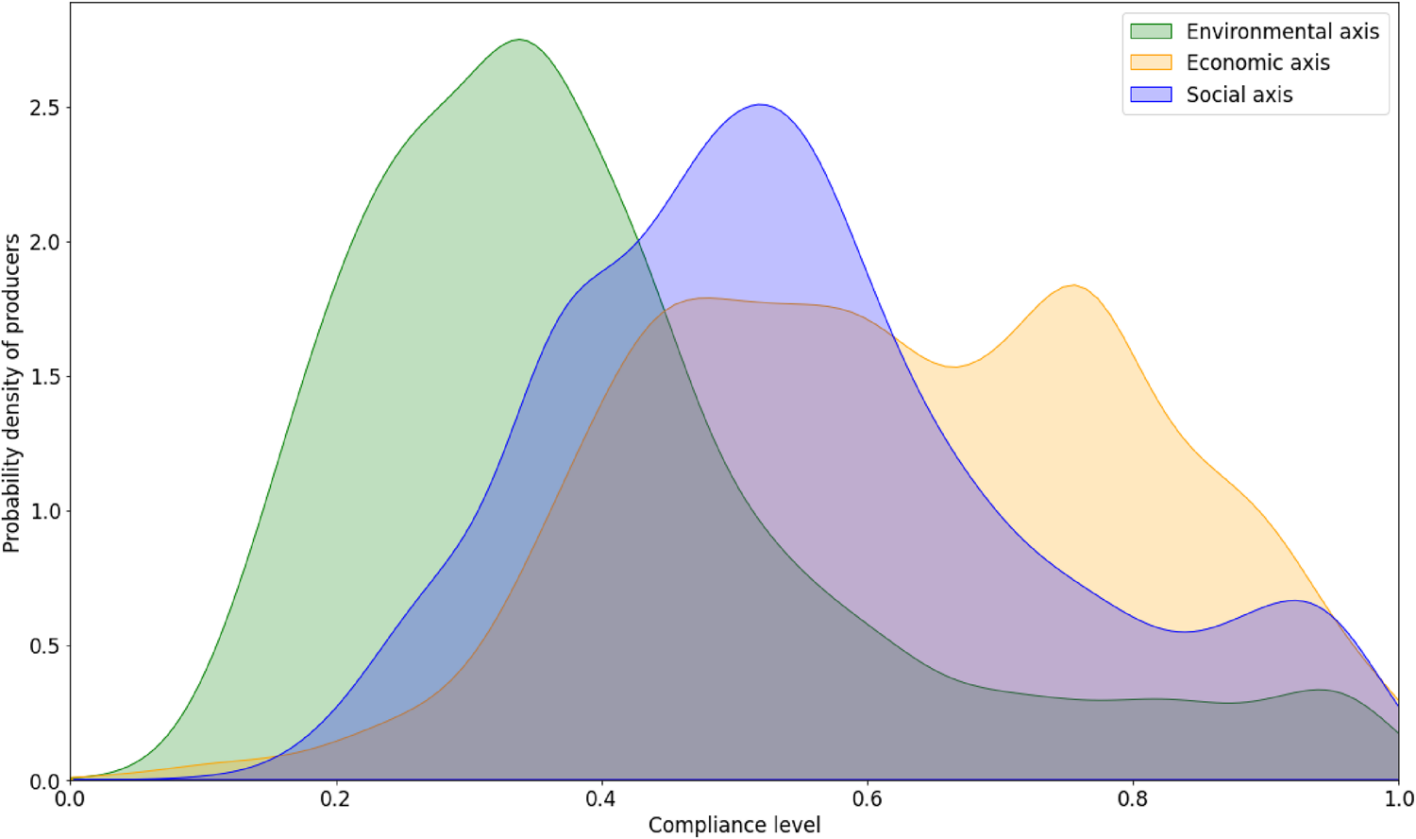
Compliance levels across sustainability axes in Colombian oil palm cultivation. This figure visualizes the distribution of oil palm producers in Colombia by their compliance with sustainable practices across three main axes: environmental, social, and economic, as assessed by the SI from 2020 to 2022^33,36^. Each axis is color-coded: green for environmental, blue for social, and orange for economic.

Identifying the gaps in SI compliance and understanding the direct and indirect factors that influence the adoption of best sustainability practices represents a significant step towards closing these gaps and achieving the desired impact at the cultivation level. However, despite the progress made, the levels of compliance across the three axes of sustainability in Colombian oil palm cultivation still show significant delays. Figure 2 shows that the greatest concentration of producers is found among those with low compliance levels in environmental practices, medium compliance in social practices, and medium to high compliance in economic practices^33,34^. This uneven distribution highlights the ongoing challenges in achieving comprehensive sustainability within the sector.

While our study does not aim to identify the reasons for sustainable technology adoption or non-adoption directly, we are contributing to the literature of agricultural extension by conducting an in-depth analysis of the SI to identify clusters of producers based on their compliance levels with economic, environmental, and social practices. Therefore, to guide this investigation, we pose two primary research questions:

- *What patterns of technological implementation can be identified within SI data?*
- Furthermore, suppose there are groups of producers that exhibit similar compliance behavior with sustainable practices. In that case, we pose a second research question: *What are the key producer typologies emerging from SI compliance data?*

This research aims to generate more effective technological extension strategies sensitive to the particularities and specificities of each identified producer group. Our findings expand the conceptual framework of agricultural extension by integrating sustainability indices and data-driven clustering approaches into extension strategy development. This novel approach contributes to a better understanding of the challenges and opportunities faced by oil palm cultivation in Colombia, moving beyond traditional categorizations based on scale, age, region, or gender of producers. To address these research questions, we employ clustering techniques, specifically K-means algorithms and Ward’s method, for data analysis. Our literature review indicates that detailed studies analyzing SI in agriculture to generate technological extension intervention strategies are scarce. Therefore, this research extends the knowledge frontier by applying sophisticated data analysis techniques to sustainability index data in the context of agricultural extension.

## Materials and methods

All methods were carried out in accordance with relevant guidelines and regulations. Ethical review and approval were not required for this study, as it involved no experiments on humans or the use of human tissue. However, all data were collected with the informed consent of participating producers, anonymized prior to analysis, and processed in accordance with Colombia’s data protection law (Law 1581 of 2012) and the guidelines of the “Manual for the Responsible Use of Information” issued by Fedepalma and Cenipalma.

### Location and context

**Fig. 3:**
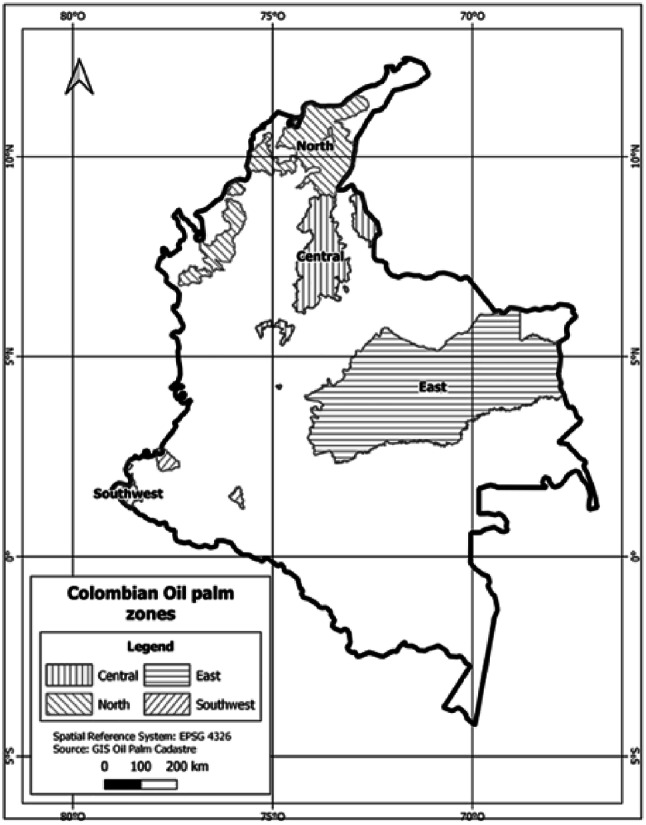
Geographic location of the four oil palm zones in Colombia. Source: Cenipalma 2023^38^. Colombia is the world’s fourth-largest palm oil producer and the leading producer in the Americas, with operations in 155 municipalities across 20 departments in the country.

The present study was conducted within the context of oil palm cultivation in Colombia, an industry that in 2023 represented 17.6% of the national agricultural GDP^35,37^. Oil palm cultivation covers 580,000 hectares of production nationwide, distributed across four palm-growing zones, each with unique characteristics that influence the sustainability of the cultivation (Fig. 3).

Colombia’s four oil palm growing zones exhibit marked environmental and socio-economic differences that influence sustainability adoption. The Northern Zone is characterized by coastal lowlands with high rainfall variability and a predominance of small and medium producers operating under historically fluctuating phytosanitary and market conditions. The Central Zone combines industrial-scale plantations with smallholders in inter-Andean valleys, supported by a stronger institutional presence and environmental regulatory agencies. The Eastern Zone consists of vast plains with highly mechanizable landscapes and medium-to-large producers, but long distances and dispersion limit technical assistance delivery. The Southwestern Zone presents the greatest environmental and social challenges, including difficult topography, historical public-order issues, and severe phytosanitary events that have reduced productive capacity. These contextual differences help shape the adoption of economic, environmental, and social sustainability practices across regions.”

As per Fedepalma’s records (2023)^33^, the production is distributed as follows: 21% in the Northern Zone, 31% in the Central Zone, 43% in the Eastern Zone, and 4% in the Southwestern Zone. These areas are recognized for their flat topography, low altitude, and climatic conditions ideal for palm cultivation. Importantly, they support the implementation of sustainable practices within the agricultural frontier, as defined in 2022 by the Rural Agricultural Planning Unit (UPRA in its Spanish acronym)^39^, without affecting national forest reserves^33^.

### Structure of the sustainability index

The SI is the cornerstone of our study, providing a comprehensive measure of sustainability in Colombian oil palm cultivation. Developed collaboratively by leading entities, including Fedepalma, Cenipalma, and APSColombia, the SI assesses the implementation of practices across three axes: economic, environmental, and social (Table 1.).

**Table 1:** Axes of the SI in Oil Palm Cultivation in Colombia^33,34,40^.

Axes of the Sustainability Index (SI)*	Description
Economic Axis	Agronomic and cultivation improvement practices for maximum economic value.
Environmental Axis	Environmental practices related to the efficient use of resources, non-deforestation, palm farming in harmony with its environment, and non-pollution.
Social Axis	Practices related to labor formalization, legal land tenure, risk mitigation associated with human rights (HR) protection, and ethical behavior.

*See Supplementary Information (Appendix A, Table A1).

Each axis incorporates a set of principles, which are further subdivided into issues and specific practices. These elements are assigned weighted scores from 0 to 100% based on their degree of compliance. These scores are integrated hierarchically, reflecting the adoption of practices from the most specific level to the general axes, allowing for an accurate evaluation of the current state of the farms in terms of sustainability^33^^34^,. See Supplementary Information (Appendix A, Table A1).

### Data collection and processing

Data was collected through the mobile application “Extension Solution,” developed by the Solidaridad Organization^36^ and adapted by Cenipalma to standardize SI measurement.

The dataset comprises 3,808 producers, representing 55.2% of total oil palm producers in Colombia, who agreed to participate in the study, signed informed consent, and were located in accessible areas. Data were collected directly in the field from 2020 to 2023 (Table 2)^40^.

**Table 2:** Total number of producers assessed using the Sustainability Index (SI) between 2020 and 2023.

Number of producers with SI	Colombian oil palm zone			
	North	Central	Eastern	Southwest
3.808*	27,2%	43,3%	13,5%	16%

*The collected dataset encompassed producers from small, medium, and large scales producers.

The collected data underwent rigorous preprocessing in compliance with the “Manual for the Responsible Use of Information from Fedepalma and Cenipalma”^41^ and Law 1581 of 2012, which regulates personal data processing in Colombia^42^. Duplicate entries were removed to maintain data integrity^43^. Additionally, producers were categorized according to relevant variables such as palm zone, age group, producer size, and gender, facilitating a more segmented and detailed subsequent analysis.

The 3,808 producers included in the dataset represent smallholders (≤ 50 ha), medium-scale producers (51–500 ha), and large-scale producers (> 500 ha), consistent with national classifications. Production systems vary from independent smallholders to producers affiliated with Palm Nuclei, which provide technical assistance and fruit commercialization services. These producers operate across heterogeneous landscapes, agronomic systems, and organizational arrangements, which influence their adoption of sustainability practices.

In Colombia, a Palm Nucleus (“núcleo palmero”) is an organizational model centered around an oil extraction mill that coordinates fruit purchasing, technical assistance, and compliance monitoring among surrounding independent producers. Palm Nuclei vary widely in their institutional strength, service provision, and capacity to promote technological adoption. Producer affiliation with a Palm Nucleus, therefore, shapes access to extension services, governance practices, and sustainability-related decision-making.

### Quantitative analysis and machine learning techniques

**Fig. 4:**
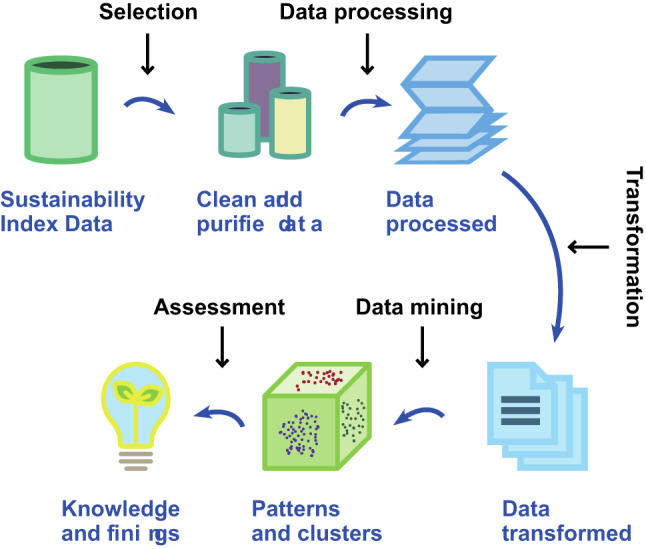
Schematic of the steps followed for data mining. Source: Adapted from Yang et al. (2020)^40,46^.

The quantitative analysis leveraged machine learning techniques, utilizing Python programming language version 3.9 and a suite of data analysis and visualization libraries, including Pandas, Numpy, Matplotlib, Sklearn, and Scipy^40^^44,45^,–^46^ (Fig. 4).

**Fig. 5:**
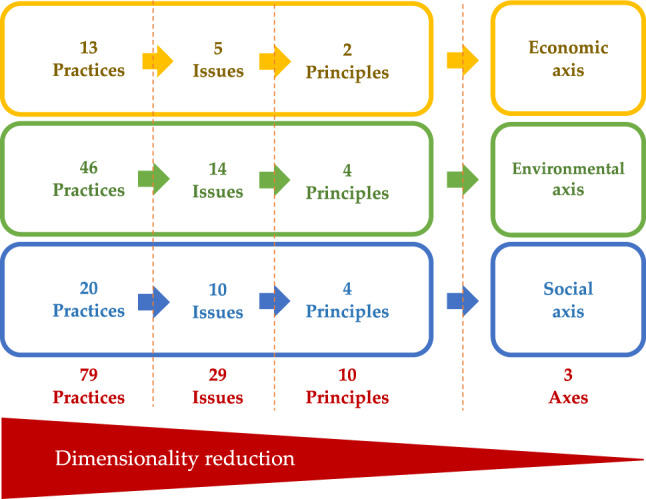
Hierarchical levels of the SI. Scheme of dimensionality reduction by integration of weighted values.

The SI’s hierarchical structure (axes > principles > issues > practices) informed our analytical approach, respecting the pre-defined weights at each level (Fig. 5). Therefore, we leveraged the high-level axes (economic, environmental, social) for data mining, as they effectively capture the overall sustainability score based on the weighted practices within the SI. These axes carry equal weight within the SI^33,34^. This method provides a more faithful representation of compliance level when implementing the evaluated practices.

### Data clustering

K-means and Ward’s clustering algorithms were employed to discern compliance patterns within the SI data^43^^47^^48^,,. This approach enabled the identification of clusters of producers with similar sustainability profiles. The optimal number of clusters at the national level was determined using the Davies–Bouldin Index and the Silhouette Coefficient, both of which indicated that six clusters maximized between-group separation while minimizing within-group variance. For regional analyses, the optimal number of clusters varied due to structural differences in the data. K-means was selected when clusters exhibited spherical distribution, whereas Ward’s method was used when hierarchical separation improved interpretability. Cluster robustness was validated through repeated initialization and consistency checks across 100 runs^43,45,49^..

Each SI practice is scored as compliant or non-compliant based on field verification by trained technicians using standardized protocols. Practice scores are aggregated into Issues, then Principles, and finally into the three sustainability axes using predefined weights established during the SI’s design and validation process. Scores for each axis range from 0% to 100%, representing the level of adoption of economic, environmental, and social sustainability practices.

We conducted an initial clustering analysis on the consolidated data of all producers at the national level. Then, we analyzed the means ($\stackrel{-}{\boldsymbol{X}}$) and standard deviations ($\boldsymbol{s}$) of each cluster for each economic, environmental, and social axes to determine the producers’ typology according to their compliance level. This analysis considered the risk level determined by Fedepalma (2023)^33^ and APSColombia (2024)^34^, which indicates that an SI $\le$ 49% constitutes a high risk (low compliance); SI between 50 and 69% constitutes a medium risk, and an SI $\ge$ 70% constitutes a low risk (advanced adoption). We then explored the characteristics of each cluster in terms of contribution by scale, palm-growing zone, age group, and gender of the evaluated producers through the analysis of Cramér’s V coefficient^50^^51^,. These thresholds were used to guide interpretation of cluster profiles but were not imposed as clustering constraints.

Given the heterogeneity of the palm-growing regions in Colombia^33^^34^,, we conducted second-level clustering analyses individually for each of the four oil palm zones in Colombia. The variable of Palm nucleus (organizational model for fruit commercialization) was added to the exploration of each cluster’s characteristics at the regional level. In this case, belonging to a specific palm nucleus can define the conditions for technology adoption. The typology of producers for each cluster was also determined following the methodology used in the national analysis. This regionalized approach allowed for greater precision in identifying compliance patterns in the SI. We selected k-means or Ward’s clustering for each zone based on achieving the least within-group variation and the most distinction between groups^43^^47^,.

### Ethics statement

This study was conducted in accordance with the Declaration of Helsinki and approved by the Institutional Research Committee of the Colombian Oil Palm Research Center – Cenipalma (protocol code 2024019000475 H, approved on 8 May 2024). The ethical review was conducted in accordance with Cenipalma’s internal research governance procedures and national guidelines for non-clinical research involving human participants in Colombia. Written informed consent was obtained from all participating producers.

## Results

The results of the clustering of oil palm producers in Colombia according to their SI are presented at the national and regional levels.

### National-level clustering

The analysis explored the correlation between categorical variables and their impact on the classification of producers. Using the K-means algorithm, chosen based on the criteria mentioned in the methodology, producers were categorized into six groups according to their levels of compliance with the SI.

**Fig. 6:**
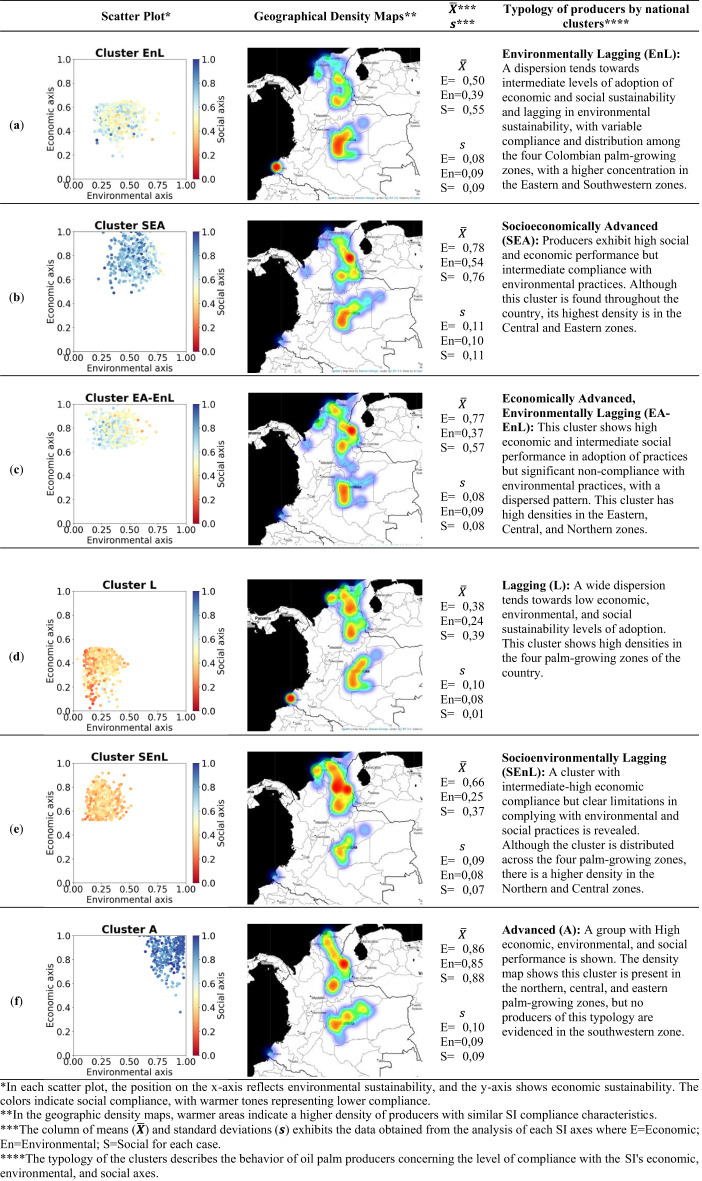
Levels of compliance and technology adoption in the economic, environmental, and social axes, along with the national geographic distribution of each cluster and its typology via SI in Colombia. **(a)** Cluster EnL; **(b)** Cluster SEA; **(c)** Cluster EA-EnL; **(d)** Cluster L; **(e)** Cluster SEnL; and **(f)** Cluster A. “The geographical density maps were generated by the authors using the Folium library for Python (version 0.16.0; Python Visualization Project, 2024; https://python-visualization.github.io/folium/latest/)”^52^.

Figure 6 displays the clustering results on scatter plots, with each point representing a distinct oil palm producer. These plots present a comparative view of producers across the SI’s economic, environmental, and social axes. This figure further shows the specific geographical distribution of each producer with SI to indicate the concentration of producers. On the left side of each subfigure, scatter plots depict the individual dispersion of producers within the axes. At the same time, the corresponding maps in the center highlight the density of each cluster. On the right, the typology of each cluster of producers is presented based on the analysis of the means ($\stackrel{-}{\boldsymbol{X}}$) and standard deviations ($\boldsymbol{s}$) according to the risk level described in the methodology. This visualization emphasized great efficiency in clustering at the high level of sustainability axes.

The six identified clusters and their characteristics are as follows (Fig. 6):

- *Environmentally Lagging (EnL)*: Producers show intermediate economic and social sustainability levels but lag in environmental sustainability. They are dispersed across all four Colombian palm-growing zones, with higher concentrations in the Eastern and Southwestern zones.
- *Socioeconomically Advanced (SEA)*: This cluster exhibits high social and economic performance but intermediate compliance with environmental practices. While present throughout the country, its highest density is in the Central and Eastern zones.
- *Economically Advanced*,* Environmentally Lagging (EA-EnL)*: These producers demonstrate high economic and intermediate social performance but significant non-compliance with environmental practices. They show high densities in the Eastern, Central, and Northern zones.
- *Lagging (L)*: This group shows low economic, environmental, and social sustainability levels. It has high densities in all four palm-growing zones of the country.
- *Socioenvironmentally Lagging (SEnL)*: These producers show intermediate-high economic compliance but clear limitations in complying with environmental and social practices. They have a higher density in the Northern and Central zones.
- *Advanced (A)*: This group demonstrates high economic, environmental, and social performance. It is present in the northern, central, and eastern palm-growing zones, but no producers of this typology are evidenced in the southwestern zone.

*In each scatter plot, the position on the x-axis reflects environmental sustainability, and the y-axis shows economic sustainability. The colors indicate social compliance, with warmer tones representing lower compliance.

**In the geographic density maps, warmer areas indicate a higher density of producers with similar SI compliance characteristics.

***The column of means ($\stackrel{-}{\boldsymbol{X}}$) and standard deviations ($\boldsymbol{s}$) exhibits the data obtained from the analysis of each SI axes where E=Economic; En=Environmental; S=Social for each case.

****The typology of the clusters describes the behavior of oil palm producers concerning the level of compliance with the SI’s economic, environmental, and social axes.

Our study did not identify, at a national level, a group that is advanced in environmental and social compliance but lagging in economic performance. Based on our experience, it is a prerequisite to achieve economic compliance first to ensure sufficient cash flow, which then allows for addressing the social and environmental axes. This is also supported by Mills et al. (2017)^53^; Kassie et al. (2015); Rogers (2010)^54^; Wejnert (2002)^55^; Bernal-Hernandez et al. (2021)^17^ who assert that access to financial capital is a key driver of technology adoption of agricultural practices.

**Fig. 7:**
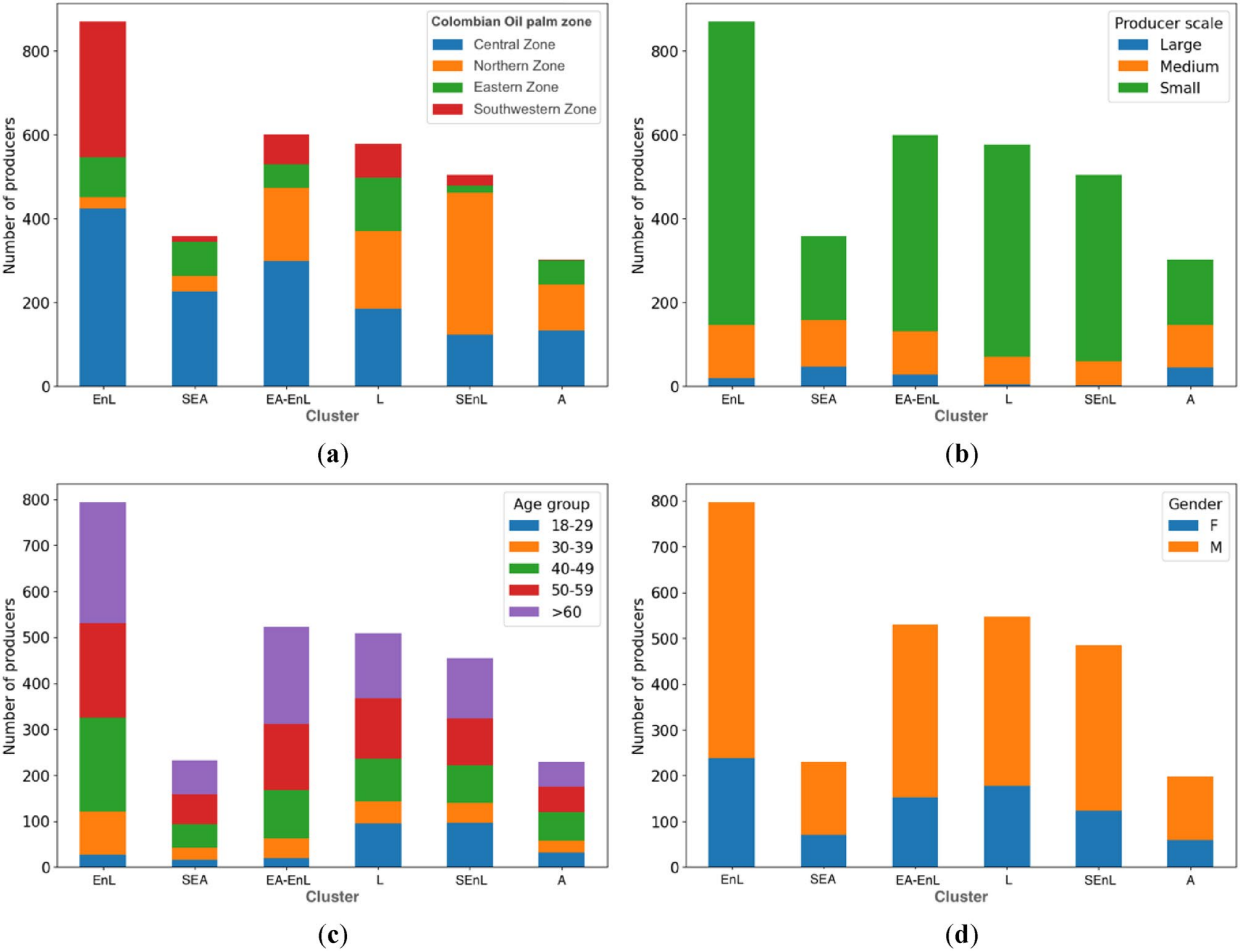
Distribution of clusters of producers, classified according to levels of SI, by (**a**) palm zone, (**b**) producer scale, (**c**) age group, and (**d**) gender.

Furthermore, the findings indicated which variables emerged as significant determinants in the classification of clusters, with the palm zone exerting the most substantial influence nationally (Cramer’s V = 46.9%), followed by the producer scale (Cramer’s V = 27.9%), and age group (Cramer’s V = 17.5%). In contrast, gender had a relatively lower impact (Cramer’s V = 10.0%) (Fig. 7).

### Regionalized clustering for each palm zone

To account for the heterogeneity observed in sustainability practices among the different palm zones, we independently conducted an additional cluster analysis for each palm zone. This differentiated methodological approach allowed for more precise identification of regional patterns and specific challenges for SI compliance, which are fundamental for developing context-adapted technological extension strategies.

We applied two different clustering techniques: K-means for the Central, Northern, and Southwestern zones, and Ward’s method for the Eastern Zone. The selection was based on achieving the least within-group variation and the most distinction between groups^43^^47^,. This stratification by palm zones and the application of selected clustering techniques allowed for a detailed assessment of compliance dynamics with practices, issues, principles, and axes of the SI in each region. As in the consolidated national analysis, the grouped results are visualized on scatter plots. See Supplementary Information (Appendix B, Fig. B1).

Table 3 shows a comparative view of the producers in the economic, environmental, and social axes of the SI in each of the oil palm zones in Colombia. This table also includes the typology of each cluster of producers and their description based on the analysis of means ($\stackrel{-}{\boldsymbol{X}}$) and standard deviations ($\boldsymbol{s}$) according to the risk level described in the methodology^33^. Additionally, Table 3 shows the percentage of producers by typology in each zone on the right. Our study explored the correlation between a set of variables (producer scale, age group, gender, and palm nucleus) and the typology of producers from the cluster analysis in each oil palm zone in Colombia, using Cramér’s V coefficient (Table 4). This statistical analysis allowed us to understand the intensity and significance of the associations between the variables and the typologies. The Palm nucleus is the organizational model for fruit commercialization in Colombia with a geographic delimitation at the subzone level, characterized by similar environmental conditions, and infrastructure for fruit commercialization where an oil mill plant aggregates palm fruit producer.

**Table 3:** Typologies of oil palm producers based on the analysis of the level of SI compliance regionalized by each palm-growing zone in Colombia.

Colombian oil palm zones (producers %)*	Number of resulting clusters (algorithm)**	Axe of SI	$\stackrel{-}{\boldsymbol{X}}$	$\boldsymbol{s}$	Typology and risk level of producers by regional clusters (abbreviation)	Producers by typology in each zone (%)***
Northern (27,2%)	6 (K-means)	E = En = S =	0,88 0,91 0,93	0,10 0,09 0,06	Advanced (A): Low risk in the three SI axes	12,2%
		E = En = S =	0,43 0,18 0,30	0,08 0,05 0,07	Lagging (L): High risk in the three SI axes	19,6%
		E = En = S =	0,84 0,56 0,68	0,09 0,08 0,12	Economically Advanced (EA): Low risk in the economic axis and medium risk in the environmental and social axes of the SI	5,4%
		E = En = S =	0,59 0,25 0,37	0,06 0,06 0,06	Socioenvironmentally Lagging (SEnL): High risk in the social and environmental axes but medium risk in the economic axis of the SI	28,6%
		E = En = S =	0,76 0,36 0,57	0,08 0,05 0,06	Economically Advanced, Environmentally Lagging (EA-EnL): Low risk in the economic axis, medium risk in the social axis, and high risk in the environmental axis of the SI	19,2%
		E = En = S =	0,77 0,28 0,36	0,06 0,09 0,06	Economically Advanced, Socioenvironmentally Lagging (EA-SEnL): Low risk in the economic axis but high risk in the environmental and social axes of the SI	15,0%
Central (43,3%)	7 (K-means)	E = En = S =	0,83 0,78 0,85	0,11 0,09 0,08	Advanced (A): Low risk in the three SI axes	11,0%
		E = En = S =	0,39 0,31 0,49	0,07 0,08 0,08	Lagging (L): High risk in the three SI axes	20,4%
		E = En = S =	0,80 0,62 0,63	0,09 0,09 0,08	Economically Advanced (EA): Low risk in the economic axis and medium risk in the environmental and social axes of the SI	9,0%
		E = En = S =	0,56 0,49 0,56	0,07 0,08 0,09	Environmentally Lagging (EnL): High risk in the environmental axis but medium risk in the economic and social axes of the SI	17,7%
		E = En = S =	0,59 0,27 0,47	0,06 0,07 0,09	Socioenvironmentally Lagging (SEnL): High risk in the social and environmental axes but medium risk in the economic axis of the SI	16,3%
		E = En = S =	0,81 0,35 0,52	0,08 0,09 0,08	Economically Advanced, Environmentally Lagging (EA-EnL): Low risk in the economic axis, medium risk in the social axis, and high risk in the environmental axis of the SI	15,3%
		E = En = S =	0,75 0,40 0,74	0,10 0,10 0,08	Socioeconomically Advanced, Environmentally Lagging (SEA-EnL): Low risk in the economic and social axes but high risk in the environmental axis of the SI	10,3%
Eastern (13,5%)	5 (Ward)	E = En = S =	0,83 0,80 0,89	0,09 0,08 0,10	Advanced (A): Low risk in the three SI axes	14,7%
		E = En = S =	0,41 0,26 0,45	0,10 0,07 0,06	Lagging (L): High risk in the three SI axes	38,5%
		E = En = S =	0,73 0,53 0,81	0,13 0,07 0,10	Socioeconomically Advanced (SEA): Low risk in the economic and social axes but medium risk in the environmental axis of the SI	14,1%
		E = En = S =	0,50 0,34 0,69	0,09 0,09 0,11	Environmentally Lagging (EnL): High risk in the environmental axis but medium risk in the economic and social axes of the SI	16,1%
		E = En = S =	0,74 0,32 0,69	0,08 0,08 0,11	Economically Advanced, Environmentally Lagging (EA-EnL): Low risk in the economic axis, medium risk in the social axis, and high risk in the environmental axis of the SI	16,6%
Southwestern (16,0%)	5 (K-means)	E = En = S =	0,36 0,36 0,40	0,09 0,05 0,06	Lagging (L): High risk in the three SI axes	13,8%
		E = En = S =	0,61 0,41 0,50	0,06 0,05 0,06	Environmentally Lagging (EnL): High risk in the environmental axis but medium risk in the economic and social axes of the SI	24,1%
		E = En = S =	0,50 0,33 0,46	0,04 0,06 0,04	Socioenvironmentally Lagging (SEnL): High risk in the social and environmental axes but medium risk in the economic axis of the SI	29,3%
		E = En = S =	0,44 0,37 0,61	0,06 0,04 0,05	Econoenvironmentally lagging (EEnL): High risk in the economic and environmental axes but medium risk in the social axis of the SI	21,3%
		E = En = S =	0,77 0,42 0,66	0,08 0,09 0,12	Economically Advanced, Environmentally Lagging (EA-EnL): Low risk in the economic axis, medium risk in the social axis, and high risk in the environmental axis of the SI	11,5%

*Each of the four Colombian palm zones is segmented, and the corresponding percentage of producers out of 3,808 producers with SI studied is shown.

**The number of resulting clusters for each regional analysis and the selected algorithm is indicated based on achieving the least variation within the group and the greatest distinction between groups.

***The percentage of producers for each typology in each palm zone is shown.

In Colombia, there are 70 palm nuclei distributed across 155 municipalities in 20 departments^35^. Belonging to a specific palm nucleus can determine how technical assistance is provided to producers to help them adopt better sustainable practices. Belonging to a specific palm nucleus may define how technical assistance is provided to producers to enable them to better adopt sustainable practices. The Palm nucleus emerged as the variable with the highest Cramér’s V for the Northern, Central, and Southwestern zones. For the Eastern Zone, both the palm nucleus and producer scale were variables with high Cramér’s V coefficients, with no significant difference between them (Table 4).

**Table 4:** Cramér’s V coefficients for the variables studied in the cluster analysis of producers with SI by Palm-Growing Zone in Colombia. The highest Cramér’s V coefficients for each palm zone are highlighted in bold.

Variables	The Cramer’s V coefficient			
	Northern	Central	Eastern	Southwestern
Producer scale	30,5%	20,7%	**45**,**5%**	26,6%
Age group	26,5%	3,5%	0,0%	4,9%
Gender	19,1%	10,0%	9,1%	3,0%
Palm nucleus*	48,1%	35,2%	43,4%	39,6%

* The Palm nucleus refers to the organizational model for fruit commercialization with a geographic delimitation at the subzone level, characterized by similar environmental conditions, and infrastructure for fruit commercialization where an oil mill plant aggregates palm fruit producer.

**Fig. 8:**
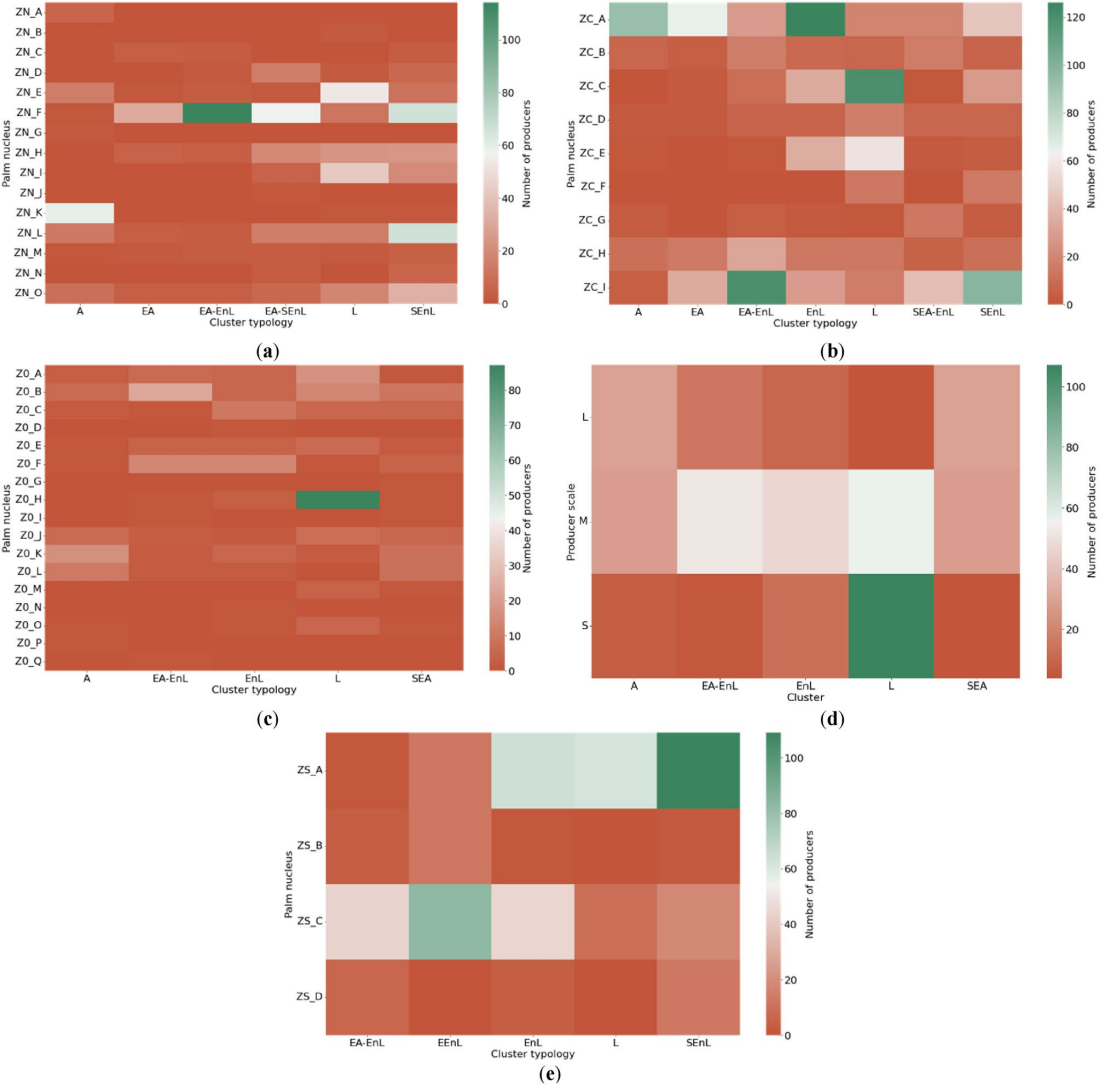
Distribution of producers by Typologies and relevant variables from Cramér’s V analysis. (**a**) Northern Zone*Palm Nucleus; (**b**) Central Zone*Palm Nucleus; (**c**) Eastern Zone*Palm Nucleus; (**d**) Eastern Zone*Producer Scale; (**e**) Southwestern Zone*Palm Nucleus.

Figure 8 provides heat maps illustrating the strongest correlations between the variables of palm nuclei and producer typologies in each palm-growing zone. This visual representation helps identify significant regional patterns and differences that reflect the contextual particularities of each zone. In each heat map, the vertical axis (y) lists the recognized palm nuclei in the corresponding zone, while the horizontal axis (x) displays the producer typologies. The density of producers in each typology is visualized through color intensity: green indicates the highest concentration of producers, and red tones indicate a lower number. An additional heat map for the “Producer Scale” variable is included for the Eastern zone, as it recorded the highest Cramér’s V value in that zone.

The following sections offer a detailed interpretation of the cluster typologies identified in each palm-growing zone of Colombia. Our analysis draws on the data presented in Tables 3 and 4 and is further informed by Cramér’s V coefficient results in Fig. 8. We focus on the “Palm Nucleus” variable for all four palm-growing zones, consistently showing strong correlations with cluster typologies. Additionally, we examine the “Producer Scale” variable for the Eastern Zone due to its high Cramér’s V value in that region. This approach allows us to highlight the highest densities of producers within each cluster, providing insights into the distribution of sustainability practices across different organizational and production scales. By doing so, we aim to uncover patterns that can inform targeted interventions and tailored extension strategies for each unique regional context.

#### Northern oil palm zone in colombia

According to the information presented in Table 3, the Northern Oil Palm Zone of Colombia represents 27.2% of the total producers evaluated for their SI. The cluster analysis revealed six distinct typologies of producers in this zone:

- ***Advanced (A)***: 12.2% of the palm oil producers in this zone are classified as advanced. These producers exhibit strong competencies in the SI’s economic, social, and environmental axes, allowing them to possibly maintain sustainable and profitable production. Their stability across all three axes suggests resilience and adaptability to regulatory or market changes without compromising long-term viability. The highest density of these producers is found in Palm Nucleus K.
- ***Lagging (L)***: 19.6% of the producers fall into this category, facing significant challenges in all sustainability axes. The combination of low economic performance, social issues, and environmental deficiencies indicates vulnerability that could hinder their long-term survival. This group likely requires intensive interventions, such as technological training and financial support, to improve their practices and reduce their dependence on external aid. The highest density is present in Palm Nuclei E, I, F, H, L, and O. Based on our experience, these palm nuclei host a larger number of smallholders who are not committed to technical assistance schemes.
- ***Economically Advanced (EA)***: This group, comprising 5.4% of producers, shows high compliance in productivity-related practices but faces moderate risks in the environmental and social axes. While they efficiently manage productivity-focused practices, there is room for improvement in social responsibility and environmental practices. Strengthening these aspects could enhance their overall sustainability and improve their image and community relations. The highest density of producers is in Palm Nucleus F.
- ***Socioenvironmentally Lagging (SEnL)***: The largest group in the Northern Zone (28.6%) corresponds to palm growers lagging in the social and environmental axes. This may indicate management practices that, while economically viable in the short term, are not sustainable and lack social correspondence. Improving these axes will be imperative for their long-term operation. The highest density of producers is in Nuclei L and F.
- ***Economically Advanced***,*** Environmentally Lagging (EA-EnL)***: Approximately 19.2% of the producers in the Northern Zone are in this category, where, despite their economic stability, they face challenges for adopting environmental and social sustainability practices. This typology highlights the need to integrate more robust and sustainable environmental practices to maintain financial viability without compromising the region’s natural environment or social fabric. The highest density is in Palm Nucleus F.
- ***Economically Advanced***,*** Socioenvironmentally Lagging (EA-SEnL)***: 15% of the producers have solid adoption of economic practices but face high risks due to non-compliance with practices associated with the social and environmental axes. This pattern indicates a disconnect between economic efficiency and socio-environmental sustainability, underscoring the importance of adopting a more integrated approach that balances all aspects of sustainable production. This cluster is unique in the Northern Zone. The highest density of producers is in Palm Nucleus F.

These findings highlight the diverse sustainability challenges in the Northern Zone, with a particular need for improvement in environmental and social practices across several producer typologies. The concentration of different typologies in specific Palm Nuclei suggests that targeted interventions at the nucleus level could improve overall sustainability in this zone. However, this poses challenges since the palm nuclei in this region may be economically depressed due to phytosanitary issues, which, according to our knowledge as of the end of 2023, devastated nearly 20,000 hectares.

#### Central Oil Palm Zone in Colombia

The Central Zone represents a significant portion of Colombia’s oil palm production, accounting for 43.3% of the producers evaluated for their SI (Table 3). Our cluster analysis revealed seven distinct typologies of producers in this zone:

- ***Advanced (A)***: 11% of producers in the Central Zone demonstrate exceptional capacity to effectively manage the economic, social, and environmental aspects of their operations. This combination of low risks across all axes suggests these producers are well-positioned to face market or agricultural changes without compromising their profitability or sustainability. The highest density of producers in this group is in Palm Nucleus A.
- ***Lagging (L)***: With 20.4% of producers classified as lagging, this zone faces significant challenges. This group may be dealing with structural problems that affect their productivity and sustainability, indicating the need for interventions not only in technological transfer but also in extension models that improve their sustainable agricultural management capacities. The highest density of producers is in Palm Nuclei C and E.
- ***Economically Advanced (EA)***: Constituting 9% of producers, this typology shows solid performance in implementing productivity-focused practices. However, they exhibit moderate risks in the social and environmental axes. Their approach could benefit from strategies that strengthen their social and environmental practices, such as closing the gaps through the implementation of strategic and operational plans for planned and sustainable technical assistance, maximizing their long-term sustainability potential. The highest density of producers is in Palm Nuclei A and I.
- ***Environmentally Lagging (EnL)***: Producers in this category (17.7%) face significant challenges in environmental management, which could compromise their future viability. This cluster requires a critical review of their agricultural practices to incorporate more sustainable methods that mitigate their environmental impact. They may need more guild support to achieve regulatory compliance. The highest density of producers is in Palm Nucleus A.
- ***Socioenvironmentally Lagging (SEnL)***: 16.3% of the producers in this zone are classified as socially and environmentally lagging, exhibiting high risks in community relations and sustainable agroecosystem management. Although the implementation of productivity-focused practices is not critical, it is not at its best performance either. Hence, improvement in all three sustainability axes is crucial for their long-term operation. The highest density is in Palm Nucleus I.
- ***Economically Advanced***,*** Environmentally Lagging (EA-EnL)***: This group, constituting 15.3% of producers, shows a disconnect between efficient economic management and environmental responsibility. These producers must adopt technologies and practices that reduce their environmental footprint to maintain competitiveness and regulatory compliance. The highest density is in Palm Nucleus I.
- ***Socioeconomically Advanced***,*** Environmentally Lagging (SEA-EnL)***: Unique to the Central Zone, this typology of producers (10.3%) represents highly efficient producers in the economic and social axes but with high risks due to non-compliance in the environmental axis. This cluster’s presence only in this zone may be due to particularities of the environment and context that should be investigated further in future studies. The highest density is in Nucleus I.

These findings highlight the diverse sustainability challenges in the Central Zone, with a particular need to improve environmental practices across several producer typologies. The concentration of different typologies in specific Palm Nuclei, especially A and I, suggests that targeted interventions at the nucleus level could be an effective strategy for improving overall sustainability in this zone. The unique presence of the SEA-EnL cluster in this zone also indicates a need for region-specific environmental management strategies. Based on our experience, this region has strengthened regional autonomous corporations that, in addition to having a regulatory role, can also provide support to producers for the implementation of sustainable practices.

#### Eastern oil palm zone in Colombia

The Eastern Oil Palm Zone constitutes 13.5% of the total sample (Table 3). Our cluster analysis, using Ward’s method, revealed five distinct typologies of producers in this zone:

- ***Advanced (A)***: 14.7% of the producers in this zone are classified as advanced, excelling in managing all critical aspects of their production. Their adaptability and sustainability are exemplary, positioning them as innovative and responsible practice leaders. The highest density of producers is in Palm Nuclei K, J, and L, particularly among medium and large-scale producers.
- ***Lagging (L)***: This is the largest cluster typology in the Eastern Zone, with 38.5%, indicating that many producers face severe limitations in managing economic, social, and environmental risks effectively. This group could benefit significantly from technological extension programs and models that involve technical assistance and training to mitigate the risks associated with the low level of SI. The highest density of producers is in Palm Nucleus H, particularly among small and medium-scale producers.
- ***Socioeconomically Advanced (SEA)***: Producers in this group, constituting 14.1%, manage economic and social aspects well but must improve environmental management. They represent a model of how economic and social practices can successfully align with market demands and community needs, although they still need to incorporate more robust environmental practices. This cluster is unique to the Eastern Zone. The highest density of producers is in Palm Nuclei B, K, and L, particularly among large and medium-scale producers.
- ***Environmentally Lagging (EnL)***: This cluster typology comprises 16.1% of the producers evaluated in the Eastern Zone and highlights the urgent need to address environmental issues that are compromising the long-term sustainability of these operations. Improving their environmental management is crucial for their survival and compliance with current regulations. The highest density of this group is in Palm Nuclei F and C, particularly among medium-scale producers.
- ***Economically Advanced***,*** Environmentally Lagging (EA-EnL)***: This group comprises 16.6% of the sample in the Eastern Zone. These producers are competent in managing productivity-focused practices that lead to economic sustainability. Still, their low performance in environmental sustainability suggests the need to integrate agricultural practices that better respect the natural environment to avoid regulatory sanctions and improve their public image. Their highest density is in Palm Nuclei B, E, and F, primarily among medium-scale producers.

A unique aspect of the Eastern Zone is the strong influence of the Palm Nucleus and Producer Scale variables, as indicated by their high Cramér’s V coefficients. This suggests that in this zone, both the organizational structure (Palm Nucleus) and the size of the operation play significant roles in access to technologies, implementation costs, and determining sustainability practices.

These findings highlight the diverse sustainability challenges in the Eastern Zone, with a particular need to improve environmental practices across several producer typologies. The large proportion of Lagging producers (38.5%) indicates a pressing need for comprehensive sustainability interventions in this zone. Based on our experience, the wide geographic dispersion in this area may limit the support and adoption of sustainable practices. However, the presence of Advanced and Socioeconomically Advanced clusters suggests that there are models of good practice within the zone that could be leveraged for peer-to-peer learning and knowledge transfer, such as planned technical assistance and monitoring of regulatory compliance by environmental and social authorities.

#### Southwestern oil palm zone in Colombia

The Southwestern Zone represents 16% of the sample studied for SI analysis (Table 3). Using the K-means algorithm, our cluster analysis revealed five distinct typologies of producers in this zone:

- ***Lagging (L)***: In the Southwestern Zone, a significant 13.8% of the producers face critical challenges in the economic, social, and environmental axes of the SI. These producers need intensive support to transform their operations into more sustainable and profitable practices, thus mitigating their dependence on external factors such as government financial support and subsidies for the removal and renewal of plantations devastated by phytosanitary issues. This behavior may result from multiple factors that have affected the palm community in this region, such as phytosanitary issues and public order problems that limit access to technologies whose implementation is measured through the SI. The highest density of producers in this category is in Nucleus A.
- ***Environmentally Lagging (EnL)***: This is the most prominent cluster typology in this zone, with 24.1%, indicating predominant environmental problems. Despite having a relatively stable economic and social base, the lack of attention to environmental sustainability could compromise their future. It is imperative that they adopt measures to improve their environmental management, for which strategies should be generated from the extension that goes beyond technological transfer and involves managing the appropriate environments and contexts to close the SI gaps. The highest density of producers is in Palm Nuclei A and C.
- ***Socioenvironmentally Lagging (SEnL)***: Representing almost a third of the producers in this zone with 29.3%, this group faces severe challenges in the social and environmental areas. Although they maintain some economic stability, improving the other two axes is imperative for long-term sustainability. Their highest density is in Palm Nucleus A.
- ***Econoenvironmentally Lagging (EEnL)***: This profile constitutes 21.3% and stands out for its difficulties in both the economic and the environmental dimensions, suggesting an urgent need to rethink their business models and agricultural practices to align them with more rigorous sustainability standards and improve their financial viability. This cluster typology is unique to the Southwestern Zone. The highest density of producers in this group is in Palm Nucleus C.
- ***Economically Advanced***,*** Environmentally Lagging (EA-EnL)***: Although these producers are economically efficient, their poor performance in environmental management constitutes a risk that must be addressed. This group comprises 11.5% of the sample studied in this zone. Integrating sustainable practices, technologies, and innovations is crucial to balance their profile and ensure a prolonged and responsible operation. Their highest density of producers is in Palm Nucleus C.

The Southwestern Zone stands out for its unique challenges, being the only region without an Advanced cluster and featuring the distinctive EEnL typology. These findings highlight the need for comprehensive sustainability interventions in this zone, emphasizing environmental practices across all producer typologies. Based on our experience, this palm-growing zone has historically faced challenges related to security, access roads to the plantations, and phytosanitary devastations. All these factors increase uncertainty, limiting producers’ decisions to adopt technologies that would help them close gaps in compliance with the economic, environmental, and social practices of the SI.

The concentration of different typologies in specific Palm Nuclei, especially A and C, suggests that targeted interventions at the nucleus level, such as strengthening the technical capacity of producers and their workers, increasing security, and improving access roads, could be an effective strategy for improving overall sustainability in this zone. However, the widespread environmental challenges across all clusters indicate that zone-wide initiatives focusing on environmental management and sustainable practices are necessary.

### Resulting typologies of oil palm producers from SI cluster analysis at regional levels

Our comprehensive cluster analysis at regional levels revealed a nuanced landscape of sustainability practices among Colombian oil palm producers based on their SI. Table 5 presents a detailed view of the distribution of 10 different typologies of oil palm producers within each palm-growing zone.

**Table 5:** Distribution of Producer Typologies according to their level of SI compliance in Oil Palm based on regional analyses in Colombia.

Typology of producers	Colombian oil palm zones			
	Northern 27,2%*	Central 43,3%*	Eastern 13,5%*	Southwestern 16,0%*
A	12,2%	11,0%	14,7%	-
L	19,6%	20,4%	38,5%	13,8%
EA	5,4%	9,0%	-	-
SEA	-	-	14,1%	-
EnL	-	17,7%	16,1%	24,1%
SEnL	28,6%	16,3%	-	29,3%
EEnL	-	-	-	21,3%
EA-EnL	19,2%	15,3%	16,6%	11,5%
SEA-EnL	-	10,3%	-	-
EA-SEnL	15,0%	-	-	-

*Each of the four Colombian palm zones is segmented, and the corresponding percentage of producers out of 3,808 producers with SI studied is shown.

Table 5 reveals how sustainability typologies vary across regions in ways that reflect territorial constraints and institutional capacities. For example, the high concentration of ‘Lagging’ and ‘Socioenvironmentally Lagging’ producers in the Southwestern Zone aligns with longstanding phytosanitary and socio-economic challenges, while the Eastern Zone exhibits stronger representation of ‘Advanced’ and ‘Socioeconomically Advanced’ groups due to higher mechanization levels and organizational stability. These results underscore the importance of regionally adapted extension strategies rather than one-size-fits-all interventions.

The typologies presented in the previous table span a spectrum of sustainability performance, from “Advanced” (A), indicating low risks across all three axes, to “Lagging” (L), facing high risks in all areas. Between these extremes, we identified more nuanced combinations such as “Economically Advanced and Environmentally Lagging” (EA-EnL) or “Socio-environmentally Lagging” (SEnL), among others, reflecting the complex interplay of sustainability factors in oil palm production.

The geographic distribution of these typologies, as illustrated in Table 5, provides a valuable tool for understanding the spatial variation of sustainability risks and capabilities across Colombia´s oil palm sector. Some typologies are unique to specific zones, highlighting the importance of regionally tailored approaches to sustainability improvement. This granular view of producer typologies across different zones offers critical insights into producers’ diverse challenges and opportunities in each region.

This detailed typology analysis has significant practical implications for Colombia’s oil palm industry stakeholders. It provides a robust foundation for organizations like the Colombian Oil Palm Research Center Corporation (Cenipalma) to design and implement targeted interventions through their technological extension teams. By understanding the specific sustainability profiles prevalent in each zone, these interventions can be tailored to address the most pressing challenges and leverage existing strengths. Importantly, this approach allows for developing strategies to promote sustainability across all producer segments, regardless of scale, gender, age group, or commercial affiliation to a Palm Nucleus.

## Discussion

The detailed analysis of the SI for oil palm cultivation in Colombia has revealed complex patterns in producers’ adoption of sustainable practices, technologies, and innovations. These findings address our first research question, confirming that it is possible to correlate SI compliance levels with technological adoption. The identified patterns underscore the inherent complexity of adopting sustainable practices, highlighting the importance of adapted and localized approaches in implementing technological extension strategies.

Our study also successfully identified ten distinct typologies of producers (1. Advanced; 2. Lagging; 3. Economically Advanced; 4. Socioeconomically Advanced; 5. Environmentally Lagging; 6. Socioenvironmentally Lagging; 7. Econoenvironmentally lagging; 8. Economically Advanced-Environmentally Lagging; 9. Socioeconomically Advanced-Environmentally Lagging; 10. Economically Advanced-Socioenvironmentally Lagging) based on their SI compliance levels, answering our second research question. This categorization of clusters provides valuable insights into the diverse sustainability challenges different producer groups face and the targets of extension strategies based on the results of most vulnerable groups.

Our findings and typologies are consistent with literature highlighting producers’ difficulties in technological adoption^14^^54^^56^,,. Bakar (2012)^8^ and Nakano et al. (2018)^9^ note that these difficulties are particularly present in developing countries, where for small-scale producers, achieving optimal levels of sustainability in agricultural practices is complex and almost impossible due to knowledge limitations, access to specialized inputs, and the need for significant investments in sophisticated equipment. Contrary to previous studies (e.g., Aguilar et al. (2013)^57^), which emphasize the role of factors such as producer scale, gender, or age in technological adoption, our findings indicate that sustainability challenges affect producers across all scales and demographic categories. This insight breaks traditional paradigms in extension strategy formulation (e.g^12,58,59^.,). It marks a new direction in creating extension models based on grouping producers by needs, particularities, and compliance levels in metrics such as the SI. While not negating the importance of scale, age, or gender, this approach complements and strengthens existing models by providing a more nuanced understanding of producer needs.

The influence of socio-economic and contextual factors is evident in the significant variation of producer densities in each cluster typology across Colombia’s palm-growing zones, indicating that specific regional factors, such as access to technology and management capacity, play a crucial role in the decision to adopt economic, environmental, and social practices framed in sustainability. This variability supports the arguments of various authors such as Acheampong et al. (2024)^22^, Martínez-Arteaga et al. (2023)^26^, Martínez (2022)^24^, Norton et al. (2020)^25^, Takahashi et al. (2020)^27^, and Loevinsohn et al. (2013)^23^ who emphasize the need for differentiated and adapted technological extension models that consider local particularities to improve the adoption of sustainable practices.

The significant influence of the Palm Nucleus variable on cluster assignment, particularly in the Northern, Central, and Southwestern zones, suggests that organizational governance plays a critical role in sustainability adoption. Stronger nuclei tend to offer structured technical assistance and clearer compliance pathways, whereas weaker nuclei operate with limited capacity to support producers. These findings align with broader literature on the role of intermediary organizations in agricultural innovation systems.

Our findings reveal challenges and opportunities in sustainability certification for Colombia’s palm oil sector. This aligns with Mwangi & Kariuki (2015)^21^, who highlight opportunities for developing countries. While the implementation of the “Palm Oil Sector Sustainability Strategy” and the “Sustainable Palm Oil Program of Colombia - APSColombia” represent significant steps towards differentiating Colombian palm oil in the global market^34^, our findings indicate that there is still a long way to go. The high proportion of producers within typologies demonstrating high or intermediate risk levels due to non-compliance with sustainable practices underscores the need for continued and intensified efforts. Nevertheless, Fedepalma et al. (2023)^35^ highlight efforts to overcome sustainability barriers by aiming at strategies that lead to market access according to European consumption trends and the effectiveness of sustainability certifications. Supporting this, our study highlights the importance of strategic interventions from the extension based on SI data to close sustainability gaps.

Using the Sustainability Index (SI) to monitor the adoption of sustainable practices, technologies, and innovations has proven valuable in identifying significant lags and risk levels across sustainability’s economic, environmental, and social axes. Importantly, it has also allowed for the identification of producer typologies with optimal compliance levels, demonstrating that sustainable production is achievable even under adverse contextual conditions^34^. Our findings underscore the need for technological extension programs that not only transfer knowledge but also facilitate the adoption of technologies and innovative practices by adapting to the specific needs of producers and managing particular contexts and environments for the social appropriation of knowledge.

The distribution of producer typologies highlights both opportunities and constraints for advancing sustainability certification. Producers with higher SI scores demonstrate greater alignment with core certification requirements, whereas typologies characterized by environmental lagging face structural challenges that hinder progress. Linking SI-based diagnostics to targeted extension strategies can support certification readiness by prioritizing environmental gaps, institutional strengthening, and technology adoption where they are most needed.

The SI’s structure aligns with several criteria found in international certification schemes, including environmental management, labor conditions, and good agricultural practices. Many SI indicators correspond directly to practices required for RSPO, Rainforest Alliance, or Fair Trade certification, making SI-based cluster profiles a useful proxy for assessing certification readiness. Additionally, high-SI producers typically exhibit greater adoption of technologies related to monitoring, record-keeping, and integrated management, all of which facilitate compliance with certification standards.

Operational and Policy Implications. The typologies derived from the clustering analysis provide actionable insights for designing targeted sustainability interventions. Public administrations can use regional typology distributions to prioritize investment in phytosanitary infrastructure, environmental monitoring, and extension coverage. Palm Nuclei can tailor their technical assistance strategies by focusing on the specific sustainability gaps of their affiliated producers. For producers, especially smallholders, the results highlight the level of commitment required to improve economic, environmental, and social performance. Tailored extension strategies differentiated by zone, producer scale, and sustainability profile can improve adoption rates and accelerate the sector’s progress toward certification and regulatory compliance.

The results of this study contribute to the conceptual framework of agricultural technological extension by providing a data-driven approach to understanding the diverse sustainability challenges faced by oil palm producers in Colombia. By identifying behavioral patterns in technology adoption based on SI data, we offer a foundation for developing more effective and sensitive extension strategies tailored to the particularities of each identified producer group.

However, it’s critical to acknowledge the limitations of our study. While our analysis provides valuable insights into sustainability adoption patterns, it does not directly investigate the causal factors behind these patterns. Future research could benefit from mixed-method approaches that combine quantitative analysis with qualitative investigations to uncover the underlying reasons for different adoption levels.

## Conclusions

Our research has demonstrated that it is possible to correlate Sustainability Index (SI) compliance levels with technological adoption in the Colombian oil palm sector, revealing complex patterns and producer typologies in implementing sustainable practices, technologies, and innovations. This finding highlights the importance of an adapted and localized agricultural extension approach that considers regional particularities and producers’ specific needs. This strategy is crucial to overcoming the barriers that hinder the widespread adoption of sustainable practices, especially in a developing country like Colombia, where knowledge and resource limitations can be more pronounced. Additionally, our results indicate that technological adoption lags are not limited to small-scale producers, as previously thought, but affect all producers, regardless of their scale, gender, or age. This conclusion challenges existing paradigms and suggests the need to reformulate technological extension strategies to focus more on the needs, particularities, and compliance levels of producers rather than solely on demographic or scale characteristics. This approach will allow for the developing of more effective and personalized extension models.

The study also shows that, although Colombia has made significant efforts to differentiate its palm oil in the global market through sustainability certifications, there are still substantial challenges to achieving and maintaining high levels of sustainable practices. The variability in the density of producers within the identified typologies demonstrates that strategies still need to be improved to close sustainability gaps and meet international standards. These efforts will improve Colombia’s position in global markets and contribute to a more sustainable industry with environmental and social responsibility while being highly productive. Our identification of ten distinct producer typologies across the national and regional levels provides a nuanced understanding of the sustainability landscape in Colombia’s oil palm sector. This categorization offers a valuable tool for policymakers, industry organizations, and extension services to tailor their interventions and support mechanisms to the specific needs and challenges of different producer groups.

Applying the SI as a monitoring tool has not only allowed the identification of lags and risk levels in adopting sustainable practices but also facilitates the identification of producer typologies that optimally comply with sustainability standards. This segmentation capability reinforces the need for technological extension programs that transmit knowledge and encourage the adoption of innovative technologies and practices, adapting to the real needs of producers to promote a more effective extension of sustainable practices.

Our findings highlight the Palm Nucleus as a key institutional actor for driving sustainability adoption. Strengthening nuclei through improved extension capacity, monitoring systems, and resource coordination may accelerate compliance with environmental and social practices, especially in zones with higher vulnerability.

Future research should focus on understanding the underlying factors that contribute to forming these different producer typologies. This could include investigating the socio-economic, cultural, and environmental factors that influence sustainability adoption at a more granular level. Additionally, longitudinal studies could provide insights into how producer typologies evolve in response to various interventions and changing market conditions.

In conclusion, this study provides a comprehensive framework for understanding and addressing sustainability challenges in the Colombian oil palm sector and possibly in other agroindustrial sectors worldwide. By offering a data-driven approach to categorizing producers based on their sustainability performance, we have laid the groundwork for more targeted and effective interventions. As the global demand for sustainable palm oil continues to grow, the insights from this research can help guide Colombia’s oil palm industry toward a more sustainable and competitive future.

## Supplementary Information

Below is the link to the electronic supplementary material.

Supplementary Material 1

## Data Availability

Data Availability Statement: The datasets analyzed in this study are not publicly available due to confidentiality agreements with participating oil palm producers and the sensitive nature of the data. However, data are available from the corresponding author upon reasonable request and subject to approval by the Colombian Oil Palm Research Center Corporation – Cenipalma.

## Abbreviations

SI: Sustainability Index
