# Supplementary material for: Compliance patterns in adopting sustainability practices: A cluster analysis of oil palm producers in Colombia
Source: Sci Rep. 2026 Mar 13;16:13354. doi: 10.1038/s41598-026-43888-9 (PMC13106722; doi:10.1038/s41598-026-43888-9)
Supplement: Supplementary file 1 — Supplementary Material 1 [file 41598_2026_43888_MOESM1_ESM.docx]

**Supplementary Material**

**Appendix A**

*Appendix A.1*

Below is the Sustainability Index (SI) for oil palm cultivation:

**Table A1**

Sustainability Index of Oil Palm Cultivation in Colombia. For each axis of sustainability, principles are presented at the first level, themes at the second level, and practices at the third level.

| **ECONOMIC AXIS** | | Weighted percentage |
| --- | --- | --- |
| **PRINCIPLE 1.** Productive, competitive, and resilient agribusiness | | 90% |
| 1.1 | Planning, design, and establishment of the plantation | 20% |
| 1.1.1. | Have studies that guarantee the proper establishment of the crop | 15% |
| 1.1.2. | Provide soil preparation according to the topography | 15% |
| 1.1.3. | Implement irrigation and drainage systems according to the needs of the crop | 30% |
| 1.1.4. | Delimit productive areas for differentiated crop management | 15% |
| 1.1.5. | Guarantee the provenance of certified planting materials | 25% |
| 1.2 | Crop maintenance | 55% |
| 1.2.1 | Keep the lot clear, facilitating maintenance tasks and transit through it | 20% |
| 1.2.2. | Design, apply, and maintain a Nutrition Plan for the crop | 50% |
| 1.2.3. | Perform integrated pest and disease management | 30% |
| 1.3 | Harvest and production | 20% |
| 1.3.1 | Harvest according to cycles, with quality criteria for harvest and bunch per cultivar | 100% |
| 1.4 | Operational management | 5% |
| 1.4.1 | Document and keep records of the tasks and procedures carried out on the plantation | 100% |
| **PRINCIPLE 6.** Value generation from biomass | | 10% |
| 6.1 | Utilization of crop residues | 100% |
| 6.1.1. | Incorporation of biomass into the crop (Mulching) | 80% |
| 6.1.2. | Utilization of other available by-products | 20% |
| **ENVIRONMENTAL AXIS** | |  |
| **PRINCIPLE 2.** Proper and efficient use of soil, water, and energy | | 25% |
| 2.1 | Soil conservation - prevention of erosion and preservation of soil ecology | 30% |
| 2.1.1. | Implement cover crops within the plantation that contribute to improving soil conditions | 30% |
| 2.1.2. | Control the spread of potentially invasive cover species | 20% |
| 2.1.3. | Use synthetic chemical products responsibly (justified and according to recommendations) | 20% |
| 2.1.4. | Do not use fire for land preparation | 30% |
| 2.2 | Water - Legal capture | 25% |
| 2.2.1. | Have the permits authorizing the capture(s) | 40% |
| 2.2.2 | Show that water consumption does not exceed the granted flow | 20% |
| 2.2.3. | Comply with all the obligations of the capture permit(s) | 10% |
| 2.2.4. | Have permits for riverbed occupation | 20% |
| 2.2.5. | Have the water concession permit updated to the required volume | 10% |
| 2.3 | Water - Efficient use | 25% |
| 2.3.1. | Use efficient irrigation systems | 40% |
| 2.3.2 | Use adequate information to decide when to irrigate/drain | 35% |
| 2.3.3. | Perform adequate maintenance of capture and distribution systems | 15% |
| 2.3.4 | Implement and monitor the water-saving and efficient use plan | 10% |
| 2.4 | Watershed management and water resource governance | 10% |
| 2.4.1. | Identify community actors who may be impacted by the plantation's water use | 40% |
| 2.4.2. | Establish and maintain communication channels with prioritized actors | 30% |
| 2.4.3 | Have active participation in governance schemes established in the watershed where it is located | 30% |
| 2.5 | Energy | 10% |
| 2.5.1. | Have records and actions to optimize fossil fuel consumption | 70% |
| 2.5.2. | Implement and monitor the energy-saving and efficient use plan (large producers) | 30% |
| **PRINCIPLE 3.** Zero deforestation and no replacement of HCV and HCS areas | | 25% |
| 3.1 | Environmental planning for no deforestation | 70% |
| 3.1.1 | Have plantations within the agricultural frontier | 35% |
| 3.1.2 | Have evidence of not replacing forests since January 2011 | 35% |
| 3.1.3 | Develop actions to restore or compensate in case of deforestation | 15% |
| 3.1.4 | Have a forest use permit in case of isolated tree felling during land preparation | 15% |
| 3.2 | Environmental planning for no replacement of High Conservation Value (HCV) and High Carbon Stock (HCS) areas | 30% |
| 3.2.1. | Have evidence of not intervening or affecting the HCV and HCS areas with the project development since 2018 | 100% |
| **PRINCIPLE 4.** Harmonious palm cultivation with its natural surroundings and biodiversity | | 25% |
| 4.1 | Plantation design and management for the protection of strategic and sensitive ecosystems | 35% |
| 4.1.1 | Respect regulatory distances for watercourse buffers, water springs, permanent wetlands, and seasonal flows | 35% |
| 4.1.2 | Protect and manage forested areas and strategic ecosystems | 35% |
| 4.1.3 | Identify/demark protection strips for water bodies and perform differentiated applications of agrochemicals | 30% |
| 4.2 | Plantation design and management for HCV protection | 30% |
| 4.2.1 | Identify areas with HCV and HCS | 25% |
| 4.2.2 | Implement a management and conservation plan for HCV and HCS areas | 50% |
| 4.2.3 | Monitor RAP species and ecosystems | 25% |
| 4.3 | Plantation design and management for adopting Landscape Management Tools (LMT) | 35% |
| 4.3.1. | Include LMT in plantation design or redesign according to property conditions | 40% |
| 4.3.2. | Implement LMT according to the design | 60% |
| **PRINCIPLE 5.** Prevention and mitigation of environmental pollution | | 25% |
| 5.1 | Wastewater management | 20% |
| 5.1.1. | Have systems for treating agrochemical wastewater | 35% |
| 5.1.2. | Have systems for treating domestic and/or industrial wastewater | 20% |
| 5.1.3. | Have permits authorizing the discharge | 30% |
| 5.1.4. | Comply with all the obligations of the discharge permit(s) | 15% |
| 5.2. | Atmospheric emissions and GHG | 10% |
| 5.2.1. | Quantify GHG emissions | 60% |
| 5.2.2. | Implement a GHG emissions reduction plan | 40% |
| 5.3. | Management of chemical and biological substances | 35% |
| 5.3.1. | Properly use, handle, and mobilize chemical and biological products and substances | 35% |
| 5.3.2. | Have properly trained personnel for the use and handling of chemical and biological substances | 35% |
| 5.3.3. | Have SOPs for the safe handling of chemical and biological substances | 30% |
| 5.4. | Management of ordinary and hazardous waste | 35% |
| 5.4.1. | Quantify and take actions to reduce the generation of ordinary and hazardous waste | 10% |
| 5.4.2. | Properly separate, segregate, and store waste according to its nature (hazardous and non-hazardous) | 25% |
| 5.4.3. | Take actions for the proper management of ordinary waste, including usable (compostable, recyclable, reusable, and/or post-consumption) | 15% |
| 5.4.4. | Deliver hazardous waste (HW) to an authorized transporter (with a license for transporting hazardous materials) and have transport manifests | 20% |
| 5.4.5. | Manage the final disposal of HW with an authorized company (with an environmental license) | 20% |
| 5.4.6. | Have certificates of final disposal of HW | 10% |
| **SOCIAL AXIS** | |  |
| **PRINCIPLE 7.** Decent employment and workers' rights | | 40% |
| 7.1 | Labor formality | 35% |
| 7.1.1 | Workers are formally linked through a contract | 40% |
| 7.1.2 | Workers receive a fair wage for their work | 25% |
| 7.1.3 | Family members working on the crop have social security | 25% |
| 7.1.4 | Foreign workers linked to the crop comply with legal conditions | 10% |
| 7.2 | No forced labor | 15% |
| 7.2.1 | The employer does not condition wage payment on fulfilling requirements that exceed the employment contract | 100% |
| 7.3 | No child labor | 15% |
| 7.3.1 | No workers below the legal age are employed according to Colombian regulations | 60% |
| 7.3.2 | Permits are obtained for employing adolescents within the permitted age | 20% |
| 7.3.3 | Family minors linked to crop tasks are under family work conditions | 20% |
| 7.4 | Occupational Health and Safety Management System (OHSMS) | 35% |
| 7.4.1 | The producer has an OHSMS according to the number of workers (direct and with contractors) | 100% |
| **PRINCIPLE 8.** Responsible supply schemes and inclusive businesses | | 10% |
| 8.1 | Supplier management | 100% |
| 8.1.1 | The producer is clear about their supplier scheme and its legal compliance | 50% |
| 8.1.2 | There are transparency conditions in the producer's relationship with suppliers | 50% |
| **PRINCIPLE 9.** Responsible relationship with communities and human rights | | 35% |
| 9.1 | Planning to mitigate risks related to land tenure | 40% |
| 9.1.1 | The producer can prove land tenure or legal use of the property | 40% |
| 9.1.2 | The producer knows if their property is located within or borders collective territories | 20% |
| 9.1.3 | The producer conducts due diligence regarding the legal use rights of the property | 40% |
| 9.2 | Planning to mitigate risks related to human rights and business protection | 30% |
| 9.2.1 | The producer knows and practices measures to guarantee respect for human rights | 100% |
| 9.3 | Planning to mitigate risks related to the social environment | 30% |
| 9.3.1 | The producer identifies relevant actors in the environment where the crop is developed | 50% |
| 9.3.2 | There are communication and management channels for stakeholder inquiries or complaints | 30% |
| 9.3.3 | The producer develops activities to positively impact community development | 20% |
| **PRINCIPLE 10.** Ethical, legal, and transparent behavior | | 15% |
| 10.1 | Ethics and good governance | 50% |
| 10.1.1 | The producer acts according to ethical principles in their business development | 100% |
| 10.2 | Risk mitigation | 50% |
| 10.2.1 | The producer seeks to minimize the risk of involvement in illegal activities | 100% |

Cenipalma extends special recognition to the researchers who have participated in the development of the Sustainability Index (SI) tool, from its inception to its maturation and implementation, for the benefit of Colombian oil palm growers. The following researchers have contributed to this development in various capacities: Carlos Andrés Rincón Molina, Silvia Juliana Ojeda Angarita, Diego Fernando Muñoz, Weesmery Navarro, Alcibiades Hinestroza, Juan Carlos Espinoza, Julián Cifuentes, Gustavo Adolfo Gómez, Carolina Obando, María Rueda, Camilo Cortes, Jorge Alonso Beltrán Giraldo, and Julián Fernando Becerra-Encinales.

**Appendix B**

*Appendix B.1*

Each scatter plot's position on the x-axis reflects environmental sustainability, and the y-axis shows economic sustainability. Colors indicate social compliance, with warmer tones representing lower compliance.

| **Scatter plots of the typology of clusters of producers in each palm oil zone in Colombia** | | | |
| --- | --- | --- | --- |
| **Northern** | **Central** | **Eastern** | **Southwestern** |
| 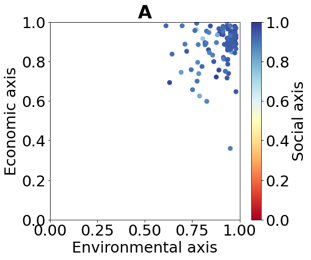 | 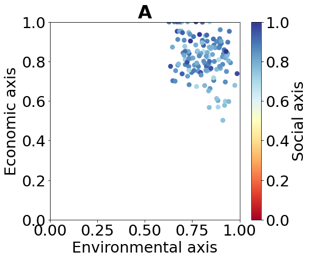 | 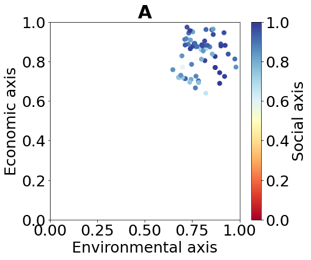 | - |
| 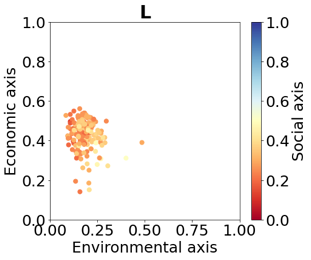 | 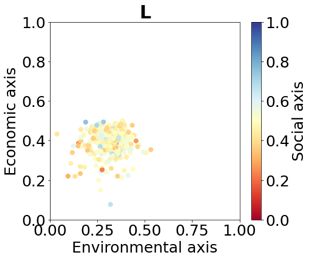 | 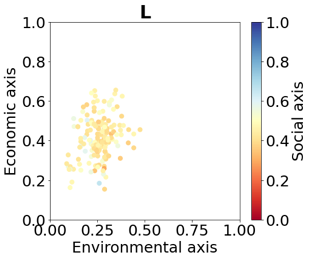 | 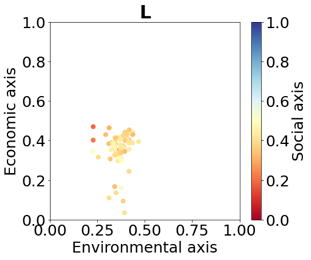 |
| 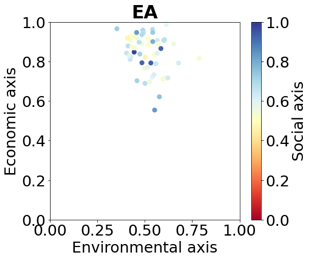 | 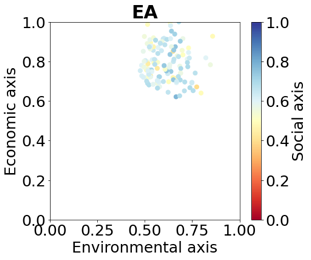 | - | - |
| - | - | 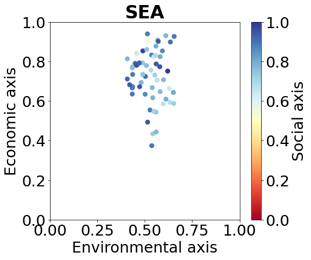 | - |
| - | 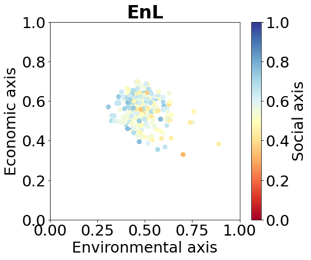 | 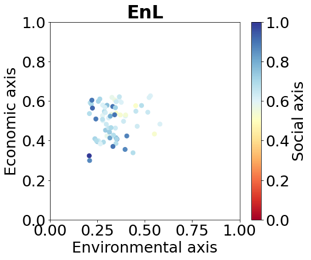 | 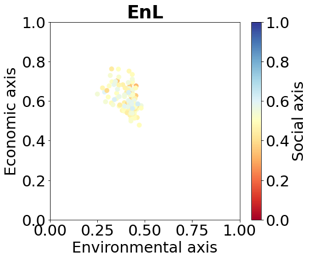 |
| 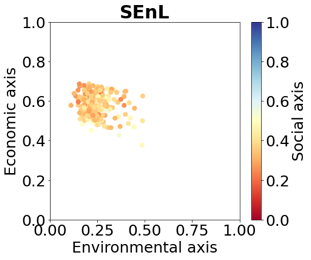 | 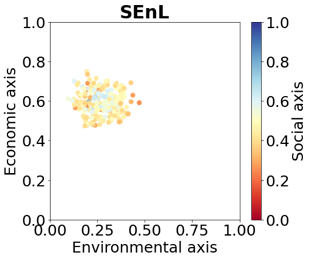 | - | 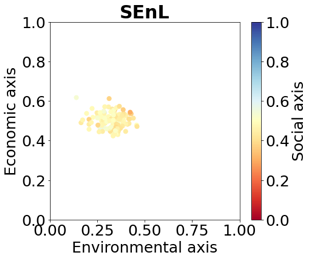 |
| - | - | - | 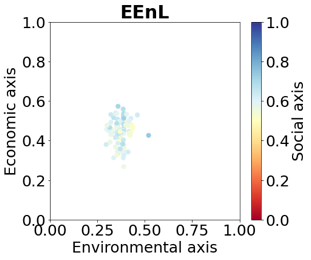 |
| 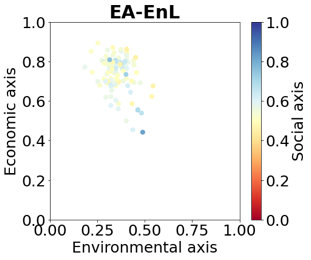 | 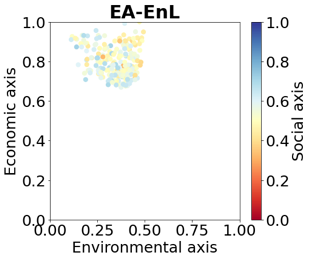 | 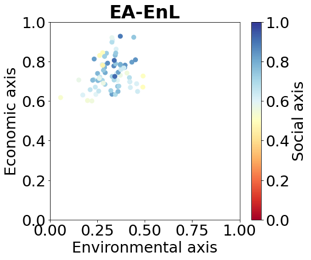 | 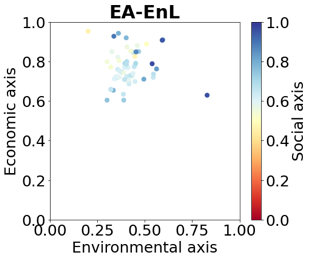 |
| - | 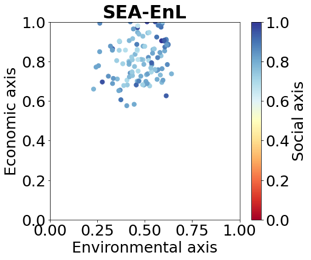 | - | - |
| 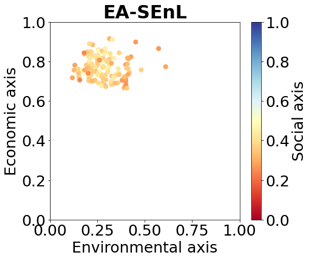 | - | - | - |

Fig. B1. Cluster analysis for each palm zone of compliance levels and adoption of technology in the economic, environmental, and social axes of the SI of the crop.
